# Precision medicine implementation challenges for *APOL1* testing in chronic kidney disease in admixed populations

**DOI:** 10.3389/fgene.2022.1016341

**Published:** 2022-12-15

**Authors:** Giovanna Câmara Giudicelli, Celia Mariana Barbosa De Souza, Francisco Veríssimo Veronese, Lygia V. Pereira, Tábita Hünemeier, Fernanda Sales Luiz Vianna

**Affiliations:** ^1^ Departamento de Genética e Biologia Evolutiva, Instituto de Biociências, Universidade de São Paulo, São Paulo, SP, Brazil; ^2^ Laboratório de Medicina Genômica, Centro de Pesquisa Experimental, Hospital de Clínicas de Porto Alegre, Porto Alegre, RS, Brazil; ^3^ Instituto Nacional de Ciência e Tecnologia de Genética Médica Populacional, Porto Alegre, RS, Brazil; ^4^ Departamento de Nefrologia, Hospital de Clínicas de Porto Alegre, Porto Alegre, RS, Brazil; ^5^ Programa de Pós-graduação em Medicina: Ciências Médicas, Universidade Federal do Rio Grande do Sul, Porto Alegre, RS, Brazil; ^6^ Institut de Biologia Evolutiva, CSIC/Universitat Pompeu Fabra, Barcelona, Spain; ^7^ Departamento de Genética, Instituto de Biociências, Universidade Federal do Rio Grande do Sul, Porto Alegre, RS, Brazil; ^8^ Programa de Medicina Personalizada Hospital de Clínicas de Porto Alegre, Porto Alegre, RS, Brazil

**Keywords:** population genomic research, genetic diversity, African ancestry, health inequities, personalized medicine

## Abstract

Chronic Kidney Disease (CKD) is a public health problem that presents genetic and environmental risk factors. Two alleles in the Apolipoprotein L1 (*APOL1*) gene were associated with chronic kidney disease; these alleles are common in individuals of African ancestry but rare in European descendants. Genomic studies on Afro-Americans have indicated a higher prevalence and severity of chronic kidney disease in people of African ancestry when compared to other ethnic groups. However, estimates in low- and middle-income countries are still limited. Precision medicine approaches could improve clinical outcomes in carriers of risk alleles in the Apolipoprotein L1 gene through early diagnosis and specific therapies. Nevertheless, to enhance the definition of studies on these variants, it would be necessary to include individuals with different ancestry profiles in the sample, such as Latinos, African Americans, and Indigenous peoples. There is evidence that measuring genetic ancestry improves clinical care for admixed people. For chronic kidney disease, this knowledge could help establish public health strategies for monitoring patients and understanding the impact of the Apolipoprotein L1 genetic variants in admixed populations. Therefore, researchers need to develop resources, methodologies, and incentives for vulnerable and disadvantaged communities, to develop and implement precision medicine strategies and contribute to consolidating diversity in science and precision medicine in clinical practice.

## 1 Background

Chronic Kidney Disease (CKD) is a worldwide public health issue with a high impact on morbidity and mortality. CKD classification is related to the disease’s repercussions on individuals’ health, resulting in a decrease in renal function without clinical manifestations until the development of end-stage renal disease (ESRD), need for dialysis, and death from cardiovascular disease (Kidney Diseases: Improving Global Outcomes—KDIGO, 2012). In Europe, the prevalence of ESRD has been estimated to be 801 patients per million (Kramer et al., 2018), while in the United States of America, it reached 2,242 cases per million in 2018 (United States Renal Data System (USRDS), 2020). In Latin America and the Caribbean, overall CKD prevalence has been estimated at 10% (Sequeiros-Buendia et al., 2021), although the prevalence rates vary among countries (Cusumano and Bedat, 2008; Luxardo et al., 2022).

Diabetes mellitus, systemic arterial hypertension, glomerulonephritis, polycystic kidneys, and medications associated with acute interstitial nephritis, such as penicillin and diuretics (Evans and Taal, 2011), are among the most common CKD causes. Nevertheless, CKD of unknown etiology ranges between 10% and 36% in adults (Taal and Brenner, 2006; Connaughton et al., 2019). Focal segmental glomerulosclerosis (FSGS) is currently the most prevalent type of primary glomerular disease as an etiologic factor of ESRD in the Americas (United States Renal Data System (USRDS), 2020; Polito et al., 2010). FSGS can be a primary or idiopathic disease or secondary to other conditions, including obesity, HIV infection, reflux nephropathy, severe systemic arterial hypertension, or reduced renal mass (Rosenberg and Kopp, 2017). Indeed, CDK has a complex and multifactorial etiology, presenting both genetic and environmental risk factors. Major risk factors associated with developing and progression of CKD are family history, socioeconomic status, African ancestry, proteinuria, obesity, and hypertension (Taal and Brenner, 2006; Weiner, 2007). Several studies have indicated a higher prevalence and severity of CKD in the people with African ancestry, pointing the need to address diagnostic and treatment strategies for earlier identification and management of the disease in this population group (Kula et al., 2022; Wilk et al., 2022).

In 2010, two studies on African Americans identified genetic variants, named G1 and G2 alleles, in the Apolipoprotein L1 (*APOL1*) gene associated with an increased risk of developing hypertension-attributed ESRD or FSGS (Genovese et al., 1979; Genovese et al., 2010). The G1 allele encompasses two nonsynonymous coding variants in the last exon of *APOL1*, rs73885319 (S342G) and rs60910145 (I384M); the G2 allele is a six base pair deletion (rs71785313) physically close to G1 allele (Genovese et al., 1979). These two alleles are common in chromosomes of individuals of African ancestry, but rare in European descent. *In vitro* assays revealed that only *APOL1* variants that were associated with kidney disease were able to lyse and provide resistance to the protozoan *Trypanosoma brucei rhodesiense*, which causes sleeping sickness in humans and primates (Genovese et al., 1979). Researchers hypothesized that G1 and G2 variants provided resistance against protozoan infections in Africans at the cost of developing kidney disease specifically in this population (Genovese et al., 1979; Beckerman and Susztak, 2018). Although the molecular mechanisms related to the increased risk of CKD are not fully elucidated, the main hypotheses investigate the potential cytotoxicity and cell death induced by *APOL1* when in the presence of these genetic variants (Friedman and Pollak, 2021). Therefore, it is hypothesized that the evolution of a critical survival factor in sub-Saharan Africa may have contributed to the high rates of kidney disease in individuals of African ancestry (for an extensive review see Daneshpajouhnejad et al. (2022).

The risk haplotype, which included the presence of both G1 and G2 alleles, has major effects on different types of non-diabetic kidney disease, such as ESRD associated with hypertensive nephrosclerosis, primary FSGS, and HIV-associated nephropathy (HIVAN). In Afro-descendants carrying G1 and G2 alleles, the increased risk of FSGS and HIVAN are, respectively, 17 and 29 times more than the non-carriers, and the disease develops at an earlier age, has a faster progression to CKD, and leads to a lower long-term renal survival (Kopp et al., 2011). The presence of G1 and G2 alleles implied a significant increase FSGS risk, with an odds ratio (OR) of 10.5 (95% confidence interval (CI) 6.0–18.4) (Kopp et al., 2011). Subsequently, they were also associated with risk for ESRD secondary to systemic arterial hypertension, with OR 7.3 (95% CI 5.6–9.5) (Freedman et al., 2009). In these initial studies, individuals with only one risk allele (G1 or G2) or no risk allele had no risk or minimal risk of kidney disease. Thus, the risk of kidney disease mediated by genetic variants in *APOL1* follows an autosomal recessive inheritance, unlike more common complex variants that follow an additive or multiplicative pattern (Friedman and Pollak, 2011).

**FIGURE 1:**
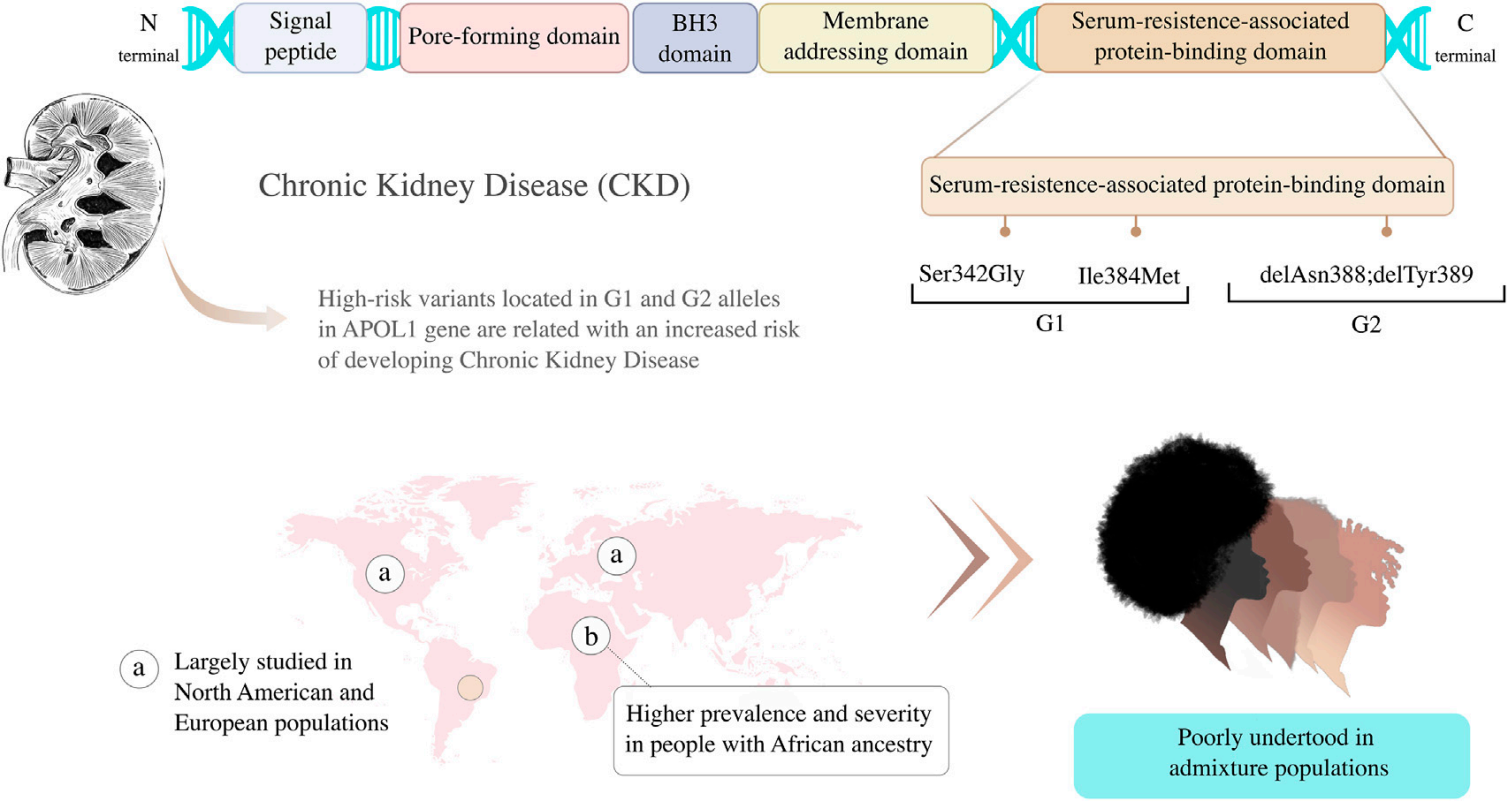
Genetic variants located in G1 and G2 alleles in Apolipoprotein L1 (*APOL1*) gene are associated with higher risk of developing Chronic Kidney Disease (CKD). Graphic representation of *APOL1* protein principal domain and variants adapted from Daneshpajouhnejad et al. (2022).

A decade after the evidence that genetic variants in *APOL1* are associated with kidney disease, much remains to be understood about the genetics and evolutionary aspects of this discovery (Adeyemo et al., 2021). Different studies have been conducted on Afro-Americans and/or Europeans concerning the impacts of G1 and G2 alleles on these populations, but other under-represented populations need to be investigated in order to include diversity in genomic research and understand the impact of the risky variants in admixed populations (Hindorff et al., 2018). Here we aim to discuss the main challenges related to *APOL1* testing in CKD in admixed populations and the opportunities to better understand how genomic studies on CKD could transform precision medicine strategies through the inclusion of genetic diversity (Table 1; Figure 1).

**TABLE 1:** Main challenges, opportunities, and perspectives of applying precision medicine for *APOL1* testing in Chronic Kidney Disease (CKD) in admixed populations.

Main challenges	Opportunities and perspectives
We do not know the actual prevalence of high-risk *APOL1* alleles in admixed populations and there is no phenotypic marker for direction precision medicine *APOL1* testing	Genomic studies in admixed populations will help to understand genetic background population
Difficulties in genetic testing in low- and middle-income countries	Create testing centers and public policies specifically for the inclusion of *APOL1* testing in the African-descendant population
There is no specific guideline or therapies for high-risk *APOL1* asymptomatic carries	Systematic reviews and evaluation of *APOL1* testing for transplant recipients and donors, and specific clinical situations

## 2 Main challenges associated with *APOL1* testing in admixed populations

The recognition of the association between *APOL1* variants and CKD in African Americans (Genovese et al., 1979; Genovese et al., 2010) was considered a promising perspective for precision medicine. However, it remains uncertain when *APOL1* genetic testing is clinically indicated. Preliminary recommendations for clinical testing have been suggested (Kopp and Winkler, 2020), but many questions need to be answered before establishing guidelines for precision medicine *APOL1* testing. Firstly, it is important to know the proportion of individuals with high-risk genotypes and how many of them could develop ESRD in the general population. Precision medicine approaches are largely skewed towards European populations, with fewer studies representing people from African, Latin American, Caribbean, and Indigenous ancestry (Depine and Martínez, 2018; Burgos-Calderón et al., 2021; Hoy and Ordunez, 2022). Many factors contribute to geneticists favoring cohorts of European ancestries, such as the previous existence of large datasets generated by well-established organizations, and the difficulties of obtaining funding for studies focused on ethnic minorities (Popejoy and Fullerton, 2016; Hindorff et al., 2018). This bias suggests that genomics is failing on diversity. In consequence, the unequal representation of genetic variability across ancestry groups is that researchers will continue to miss important associations between variants and diseases, as well as between variants and responses to drugs (Petrovski and Goldstein, 2016; Sirugo et al., 2019).

The unbalanced representation in the population genomics research is particularly worrying for CKD since the disease is more prevalent in people of African ancestry. Another concerning point is the insufficient evidence of phenotypic markers that could guide clinical genetic testing for *APOL1* in admixed populations (Lopez-Giacoman and Madero, 2015; Cañadas-Garre et al., 2019). George et al. (2018) evaluated studies about the genetic risk of CKD inferred by genetic variants among African populations living in Africa. They suggested that studies reporting the association of genetic variants with prevalent CKD, ESRD, or CKD-related traits were inconclusive. Aiming to answer questions related to genetics and CKD, studies applying next-generation sequencing approaches have been conducted (Groopman et al., 2019; Mann et al., 2019; Rasouly et al., 2019; Breeze et al., 2021). Common indications for CKD genetic testing include unclear etiology, positive family history, early-onset, and personalized treatment based on genetic biomarkers (Devarajan et al., 2022). Examples of the clinical utility of genetic diagnoses in nephrology have been discussed (Groopman et al., 2018), ratifying the importance of a specific marker to orientate precision medicine *APOL1* testing. Also, it has been pointed out the need to include more African Americans in genomic research (Young et al., 2017; George et al., 2018). Thus, it is also essential to incorporate African descendants from admixed populations, especially from low- and middle-income countries, as well as admixed populations, where the prevalence and impact of the variants on disease could be different compared to other studies.

Actually, not only the lack of genomic studies is worrying in individuals with African Ancestry, but also studies on CKD prevalence in people Latin American and Indigenous people, especially in low- and middle-income countries (Popejoy and Fullerton, 2016). Latin America is a region profoundly affected by inequities that present an increase of prevalence concern with CKD and ESRD progression. Although there are ongoing or in development national detection programs for CKD in some Latin American countries, studies in this region have been mainly conducted using few subjects from very specific populations (Sequeiros-Buendia et al., 2021). The prevalence rate of ESRD differently varies among countries in Latin America, but it has been growing in all of them over the past years (Luxardo et al., 2022). This occurs for many reasons, including the increase in life expectancy, the type 2 diabetes epidemic, and lifestyle changes (Cusumano and Bedat, 2008). In Brazil, for example, the last Dialysis Census-estimated CKD prevalence and incidence of 640 per million population (pmp) and 204 pmp, respectively (Neves et al., 2020). This Census has also revealed an estimated annual crude mortality rate of 19.5%, suggesting a great impact of CKD on Brazilian population health. Interestingly, the Brazilian Dialysis Registry published data for the period between 2000 and 2012, in which 40.5% of people in a chronic dialysis program declared themselves to be of African descent. Despite the high prevalence and mortality of CKD in Brazil, there are no robust studies regarding the prevalence and impact of variants in *APOL1* in that population. Thus, large-scale genetic studies are required to better understand the susceptibility and severity of *APOL1* variants in admixed populations with higher African ancestry.

It should also be noted that although the presence of the high-risk genetic variants increases the susceptibility to several kidney diseases, most individuals with *APOL1* high-risk variants will not develop kidney disease, harming the ability to predict the patient’s lifetime risk (Kopp and Winkler, 2020). Thus, there are barriers to optimal kidney care related to standardization of when and how to manage people carrying high-risk variants in *APOL1*. Professional societies have not yet published guidelines for clinical testing of *APOL1* kidney risk haplotype. However, as data from multiple studies have shown that *APOL1*-associated risk acts at the kidney level, the first strategy for standardization could include *APOL1* testing in the kidney transplantation setting (Kopp and Winkler, 2020). Indeed, *APOL1* testing has been proposed as part of kidney transplant protocols for both transplant patients and potential living related donors, although unclear clinical patterns of the genetic variants usually create confusion and stigma (Young et al., 2017). This happens because the effect of the high-risk *APOL1* genetic variants in kidney transplants is still unclear. The study conducted by Reeves-Daniel and colleagues (Reeves-Daniel et al., 2011) suggested that receipt of a kidney from a donor positive for *APOL1* high-risk genotypes was associated with decreased survival of the kidney. The authors showed that 50% of the recipients of donor kidneys with both *APOL1* risk variant alleles survived after 7 years, while for *APOL1* non-risk this proportion was 75% (Hazard Ratio, HR = 3.84; *p* = 0.008). However, another study revealed no differences in kidney survival 5 years after transplant, adjusting for kidney disease type, but no information on recipient genotypes was available (Lee et al., 2012). KDIGO 2017 guidelines pointed out that kidneys from lifeless African American donors that present a relative risk 2-to-4 times higher of losing the transplanted kidney when compared to individuals with zero or one *APOL1* high-risk variants (Lentine et al., 2017). More recently, Zhang and colleagues (Zhang et al., 2021) evaluated two large cohorts and showed a strong correlation between the number of recipients of high-risk alleles and death-censored allograft loss, independent of donor *APOL1* genotype and recipient ancestry (HR = 2.14; *p* = 0.006). In this regard, genetic information and diagnosis are helpful to guide and manage the transplant for both living donors and recipients, as well as contribute to therapeutic decisions when a kidney transplant is not an option (Devarajan et al., 2022).

## 3 Opportunities and perspectives

*APOL1* genotyping could be helpful and clinical testing is recommended in different clinical conditions, such as kidney transplant patients, living donors, HIV-infected patients, FSGS, lupus nephritis, and interferon treatment candidates (Kruzel-Davila et al., 2017; Kopp and Winkler, 2020). KDIGO 2017 guidelines (Lentine et al., 2017) suggested *APOL1* genetic testing specially for candidate kidney donors. Since only a proportion of individuals that present *APOL1* risk genotypes develops kidney disease, studies considering long-term outcomes of individuals with *APOL1* risk genotypes and allograft survival from donors with the *APOL1* risk genotypes will be useful to guide *APOL1* genetic testing and understand the status of *APOL1* risk variants in nephrology (Young et al., 2017). Although no specific mechanism of kidney disease association, prevention, or therapy has been yet developed for CKD, there are many examples of the clinical utility of genetic diagnoses for kidney diseases (for more information see the reviews Devarajan et al. (2022); Groopman et al. (2018). Potential therapeutic drugs and strategies have been studied (Kruzel-Davila et al., 2017), but mainly on North American and/or European populations. Those strategies include the electronic diagnosis through a computerized database that identifies patients at CKD risk and guides testing, diagnosis, and disease management (Pefanis et al., 2018), and the freely available mobile phone CKD-GO! App, which provides the CKD Management in Primary Care handbook and allows personalized action plans based on simple kidney measures (Bello and Johnson, 2022). All those strategies have not been validated in admixed populations such as the Brazilian.

A representative percentage of the cause of death worldwide is caused by CKD and other chronic non-communicable diseases, which also represent a significant healthcare cost for countries. Since most individuals affected by CKD are managed in the primary care system without consulting a nephrologist, it is essential to fulfilling the knowledge gap of primary care providers (Bello and Johnson, 2022). It must also be considered the importance of establishing centers for *APOL1* testing and public policies properly for this end, especially for underrepresented populations from low- and middle-income countries. This approach allows the detection of early diagnosis and therefore contributes to disease prevention and reduces its progression rate (Cusumano and Bedat, 2008). To provide appropriate renal care and minimize the relationship between CKD and underrepresented communities, is important to reinforce community outreach and support, providing better education, economic opportunities, and access to preventive medicine mostly for individuals with the highest risk to develop the disease (Garcia-Garcia et al., 2015).

Global genomics research made progress over the last years to resolve the lack of diversity in genomic studies, but still, more is necessary (Atutornu et al., 2022). The importance of including individuals with African ancestry that live in Africa or from admixed populations in CKD studies has been debated (Rotimi et al., 2017; Young et al., 2017), especially because there is already evidence that measuring genetic ancestry improves clinical care for admixed populations (Udler et al., 2015; Horowitz et al., 2016; Popejoy et al., 2018). Current ancestry metrics statistics are applied to correct the effect of shared ancestry on the results of association studies, and these methods are not appropriate for genome groups that are a mosaic of fragments from different population ancestry (Bustamante et al., 2011). To improve that, reference genomes from relevant ancestral populations are necessary to help researchers to distinguish spurious from real results (Bustamante et al., 2011; Bentley et al., 2017). In this sense, financial agencies should prioritize grant requests from studies with diverse cohorts (Atutornu et al., 2022), encompasses admixed populations and individuals of African ancestry (Popejoy and Fullerton, 2016). Researchers in developed countries should be encouraged to enlarge the number of individuals from minority populations in their studies, as well as investigators from developing countries must not contribute only by providing samples (Bustamante et al., 2011; Bentley et al., 2017). Implementing a global scale for population-based genomic studies demands first establishing a praxis and requires empowering physicians from low- and middle-income countries through the development of local expertise, resources, and technology centers (Bustamante et al., 2011; Popejoy and Fullerton, 2016). This also demands an integrated approach combining mass media campaigns, and interventions with primary health care services (Dorgelo and Oostrom, 2022). Some strategies have been developed. In Argentina, The National Genomics Data System of Argentina (*Sistema Nacional de Datos Genómicos*, SNDG) aims to implement the basis for genomic medical studies in the country and contribute to data sharing (Vishnopolska et al., 2018). The Brazilian Initiative on Precision Medicine (BIPMed) project aims to accelerate the implementation of precision medicine in Brazil through the incorporation of Brazilian individuals into public genomic databases (Rocha et al., 2020). The initiative “*DNA do Brasil*” aims to sequence 40 thousand Brazilian genomes from different regions of the country to better characterize this population from a genetic point of view and create a reference genome for Brazilians (Patrinos et al., 2020). These initiatives offers an opportunity to investigate the genetic determinants of CKD in African descendants from a genomic perspective, allowing us to understand the impact of both high and low penetrance variants, frequent or rare in this population, contributing to a comprehensive assessment of the genetic variability that predisposes, protects, or aggravates this disease. Similar projects should be encouraged to increase the number of studies including non-European individuals, therefore correcting the bias in genomics population studies, and contributing to consolidating diversity in science and precision medicine in clinical practice (Popejoy and Fullerton, 2016; Denny and Collins, 2021). Thus, to optimize the development and implementation of precision medicine strategies before we need knowledge of the genetic background of the studied population.

## 4 Conclusion

The World Kidney Day 2022 theme is “*Kidney health for all. Bridge the knowledge gap to better kidney care*” and the Global Strategy on Human Resources for Health: Workforce 2030 (World Health Organization, 2030) highlights the necessity of developing primary care strategies to address chronic diseases. In pursuing these goals, investigators need to develop resources, methodologies, and incentives for vulnerable and neglected communities, especially from admixed populations. CKD occurs about four to five times more often in African descendants when compared to other ethnic groups, although an estimate of the incidence of specific CKD in the African descendant population is not known in many low- and middle-income countries. This knowledge gap interferes in the planning of specific strategies and in reducing the impact of genetic variants, which can be crucial for the prevention, monitoring, and improvement of life quality of these patients and their families. Studies on multi-ethnic and mixed populations with high African ancestry, offer a relevant opportunity to identify and stratify the different risk factors of this population for developing CKD. It also contributes to the development of personalized medicine strategies on a population level and decreases health inequities in the medical system.

## Data Availability

The original contributions presented in the study are included in the article/Supplementary Material, further inquiries can be directed to the corresponding author.
